# Effect of sodium‐glucose cotransporter 2 inhibitors on the rate of decline in kidney function: A systematic review and meta‐analysis

**DOI:** 10.1111/1753-0407.13348

**Published:** 2023-01-06

**Authors:** Yanbei Duo, Junxiang Gao, Tao Yuan, Weigang Zhao

**Affiliations:** ^1^ Department of Endocrinology, Key Laboratory of Endocrinology of Ministry of Health, Peking Union Medical College Hospital, Chinese Academy of Medical Science and Peking Union Medical College Beijing China

**Keywords:** kidney function, meta‐analysis, SGLT‐2 inhibitors, systematic review, 肾功能, meta分析, SGLT‐2抑制剂, 系统综述

## Abstract

**Aim:**

To investigate the influence of sodium/glucose cotransporter‐2 inhibitors (SGLT‐2i) on renal function during the course of its administration, particularly in the initial weeks.

**Materials and Methods:**

Randomized controlled trials (RCTs) related to SGLT‐2i were searched in databases (MEDLINE, EMBASE, and Cochrane Central Register) from the database's inception to August 31, 2021. All RCTs reported the kidney outcomes of SGLT2i versus active or placebo control were included, regardless of the presence of diabetes in the patients and the baseline estimated glomerular filtration rate (eGFR). The Cochrane Collaboration risk of bias tool was used to assess the quality of the included studies. All outcome comparisons were performed using the RevMan 5.4 software.

**Results:**

Eleven RCTs with 58 534 participants reporting prespecified renal outcomes were identified. There was no heterogeneity in the baseline eGFR and urine albumin‐to‐creatinine ratio in the included studies. In the initial 2–4 weeks, there was an acute decline of eGFR in the SGLT‐2i group compared with placebo group (weighted mean difference [WMD] −3.35 ml/min/1.73 m^2^; 95% CI, −3.81 to −2.90; *I* ^2^ = 35%, *p* = .15); When compared to baseline eGFR in the SGLT‐2i group, the WMD was −4.02 ml/min/1.73 m^2^ (95% confidence interval [CI], −3.61 to −4.44; *I* ^2^ = 0%, *p* = .45). The renoprotective effect gradually appeared, and the decline rate of eGFR in the SGLT‐2i group was sustained slower than placebo. However, the statistically significant benefit of SGLT‐2i did not appear until the 104th week (the second year) (WMD 0.35 ml/min/1.73 m^2^, 95% CI, 0.04 to 0.66; *I* ^2^ = 45%, *p* = .08). Subgroup analysis showed SGLT‐2i had a similar benefit on renal function regardless of baseline eGFR values.

**Conclusion:**

SGLT‐2i consistently slowed the deterioration of eGFR since the early stage of administration, even in patients with chronic kidney disease. However, there was an acute decline in eGFR in the initial 2–4 weeks; afterwards the renoprotective effect of SGLT‐2i gradually appeared and remained stable in the next few years.

## Graphical abstract

**Highlights**

- Sodium/glucose cotransporter‐2 inhibitors (SGLT‐2i) showed an important renoprotective effect on sustained administration, regardless of the presence of diabetes, the baseline estimated glomerular filtration rate (eGFR) and different types of SGLT‐2i.
- There was an acute decline of eGFR in the initial administration (4.02 ml/min/1.73 m^2^), and renoprotective effect of SGLT‐2i appeared subsequently, which should be noticed by clinicians.

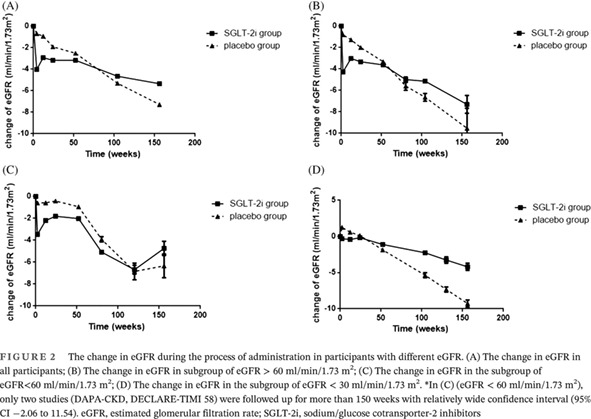

## 摘要

**目的:** 探讨SGLT‐2抑制剂(SGLT‐2i)在给药过程中, 特别是最初几周对肾功能的影响。

**材料和方法:** 从数据库创建至2021年8月31日, 在数据库(MEDLINE、EMBASE和Cochrane Central Register)中搜索与SGLT‐2i相关的随机对照试验(RCTs)。所有纳入的RCT研究都报告了SGLT‐2i组与安慰剂组的肾脏结局, 无论患者是否存在糖尿病和基线估算肾小球滤过率

(eGFR)。本研究采用Cochrane风险偏倚评估工具来评估纳入研究的质量。所有结果比较均使用RevMan 5.4软件进行。

**结果:** 共纳入11项RCT研究, 包含的58534名参与者报告了预先指定的肾脏结果。在纳入的研究中, 基线eGFR和UACR没有异质性。在最初的2‐4周内, 与安慰剂组相比, SGLT‐2i组eGFR快速下降( 加权均数差(WMD) ‐3.35ml/min/1.73m^2^; 95%CI,‐3.81~‐2.90; I^2^=35%, P=0.15);与SGLT‐2i组的基线eGFR相比, WMD为‐4.02 ml/min/1.73m^2^ (95%CI, ‐3.61~‐4.44; I^2^=0%, P=0.45)。肾保护作用逐渐显现, SGLT‐2i组eGFR下降速度持续慢于安慰剂组。然而, SGLT‐2i直到第104周(第二年)才出现统计学上显著的益处(WMD 0.35ml/min/1.73m^2^,95%CI, 0.04~0.66; I^2^=45%, P=0.08)。亚组分析显示, 无论eGFR基线值如何, SGLT‐2i对肾功能具有相似的益处。

**结论:** SGLT‐2i早期就能持续减缓eGFR的恶化, 即使在慢性肾病患者中也是如此。然而, 在最初的2‐4周内, eGFR快速下降, 之后SGLT‐2i的肾脏保护作用逐渐显现, 并在接下来的几年保持稳定。

## 1 INTRODUCTION

Sodium‐glucose cotransporter‐2 inhibitors (SGLT‐2i) are a novel class of antidiabetic drugs that promote urinary glucose excretion by reducing glucose reabsorption in renal tubules; increased urinary glucose excretion also leads to increased urinary sodium excretion.^1^, ^2^, ^3^ In recent years, it has been found that SGLT‐2i can not only reduce the blood glucose levels but also lower blood pressure, decrease uric acid and albuminuria levels, and have direct hemodynamic effect on the kidneys.^4^, ^5^ SGLT‐2i affect systemic hemodynamics, and several large‐scale clinical trials have confirmed the benefits of SGLT‐2i on the cardiovascular system and the kidneys. The Canagliflozin CardioVascular Assessment (CANVAS) study confirmed the benefit of canagliflozin on cardiovascular and renal outcomes in patients with type 2 diabetes mellitus (T2DM), and the results were consistent in people with chronic kidney disease (CKD) and with preserved kidney function.^6^ Similarly, the Empagliflozin Cardiovascular Outcome Event Trial in Type 2 Diabetes Mellitus Patients (EMPA‐REG) study reported that empagliflozin significantly decreased the risk of three‐point major adverse cardiovascular events and renal replacement therapy. The results did not differ among the subgroups based on estimated glomerular filtration rate (eGFR).^7^ Because of the benefits and safety of SGLT‐2i for treating cardiovascular and kidney disease, it has been recommended in patients with T2DM with arteriosclerotic cardiovascular disease (ASCVD) and CKD.^8^

End‐stage kidney disease is a leading cause of morbidity and mortality worldwide. A systematic review demonstrated that ~2.6 million people had received renal replacement therapy worldwide in 2010, and this number has been projected to increase to more than 5.4 million people by 2030.^9^ Several studies have confirmed the beneficial effects of SGLT‐2i on the kidney function, even in patients without T2DM. In the Multicenter Trial to Evaluate the Effect of Dapagliflozin on the Incidence of Cardiovascular Events (DECLARE‐TIMI58) study, dapagliflozin was shown to prevent the progression of kidney disease, accompanied by a reduction of end‐stage renal disease and associated mortality.^10^ The EMPagliflozin outcomE tRial in Patients With chrOnic heaRt Failure With Reduced Ejection Fraction (EMPEROR‐Reduced) study reported that empagliflozin could slow down the annual rate of decline of eGFR in patients with CKD compared with the placebo group, and the study participants were not restricted to those with diabetes.^11^ In 2019, a meta‐analysis of four large‐scale clinical trials (EMPA‐REG, CANVAS, DECLARE‐TIMI 58, and Computed TomogRaphic Evaluation of Atherosclerotic DEtermiNants of Myocardial IsChEmia [CREDENCE]) confirmed that SGLT‐2i could reduce the risk of chronic dialysis, kidney transplantation, or death due to kidney disease in individuals with T2DM.^12^

Therefore, SGLT‐2i can slow down the deterioration of renal function, even in people without diabetes mellitus. However, various mechanisms of SGLT‐2i can lead to an acute decrease in eGFR at the start of the administration, including volume depletion, vasoconstriction, excessive diuresis, and urinary tract infections, which cause concerns in patients with CKD.^13^, ^14^ In 2016, the US Food and Drug Administration issued a warning about the risk of acute kidney injury (AKI) associated with SGLT‐2i,^15^ with possible mechanisms of AKI due to hypovolemia, excessive decline in transglomerular pressure, and induction of renal medullary hypoxic injury.^16^ However, recent studies showed that the use of SGLT‐2i does not increase the risk of AKI.^17^, ^18^ Although the opinion on whether SGLT‐2i can cause AKI is not unified, SGLT‐2i does lead to an acute decline in eGFR in the initial administration.^6^, ^10^ Therefore, it is necessary to know the degree and duration of the initial decline in eGFR during therapy with SGLT‐2i, especially in patients with renal impairment.

Here, we performed a systematic review and meta‐analysis of randomized controlled trials (RCTs) to assess the effects of SGLT‐2i on renal function during both initial and long‐term administration, regardless of the presence of diabetes in the patients and the baseline eGFR.

## 2 METHODS

This systematic review and meta‐analysis was performed based on a prespecified protocol designed by the authors (Appendix S1). Databases (MEDLINE, EMBASE, and Cochrane Central Register) were searched from their inception to August 31, 2021. We included RCTs involving human participants and excluded nonrandomized trials, trials that lacked placebo or control groups, animal studies, and in vitro studies. The protocol was registered in the International Prospective Register of Systematic Reviews (PROSPERO) before the analyses were completed (PROSPERO registration number CRD42021276996). The results are reported according to the Preferred Reporting Items for Systematic Reviews and Meta‐Analysis (PRISMA) statement (Appendix S2).^19^

Two authors independently screened the titles and abstracts of all the initially identified studies. First, potential studies were screened based on titles and abstracts. We eliminated duplicate publications from the original RCT and screened the titles and abstracts. Full texts were retrieved from studies that satisfied all the selection criteria. We selected RCTs that reported the following outcomes: changes ineGFR in the initial stage and long‐term changes in the urine albumin‐to‐creatinine ratio (UACR) after medication. The inclusion and exclusion criteria for this meta‐analysis are provided in Appendix S1. Pooled or secondary analyses were included if they provided additional information about renal function beyond that found in the original RCT articles. Any disagreements related to the identification or eligibility of studies were resolved through discussion with a third author. Study sponsors and investigators were contacted to obtain additional data if necessary.

Data were extracted by two authors independently according to the specified protocol. Any discrepancies were resolved through consultation with a third author. Renal outcomes were the changes in eGFR after the therapy with SGLT‐2i. The details of the extracted information are provided in Appendix S1. We used image extraction software to extract the data presented only in figures without corresponding numerical data (WebPlotDigitizer, version 4.5). If two different doses of SGLT‐2 inhibitors were used in the original study, we chose the group using the higher dose. Subgroup analyses were performed (based on different CKD grades, patients with or without diabetes and types of diabetes, patients with or without heart disease and different types of SGLT‐2i) to explore the source of heterogeneity. The Cochrane Collaboration risk of bias tool was used to assess the quality of the included studies.^20^

### 2.1 Data synthesis and analysis

All outcome comparisons were performed using the Review Manager software (Cochrane RevMan 5.4). For continuous data, weighted mean differences (WMDs) with 95% confidence intervals (CIs) were used to assess the effect size. For discontinuous data, the relative risks (RRs) with 95% CIs were used to estimate the effect size. Heterogeneity among studies was assessed using *I* ^2^ statistics (significant for *I* ^2^ > 50%) and fixed‐effect model was adopted regardless of *I* ^2^. Statistical significance was set at a *p* value <.05.

## 3 RESULTS

### 3.1 Characteristics of included studies

**FIGURE 1:**
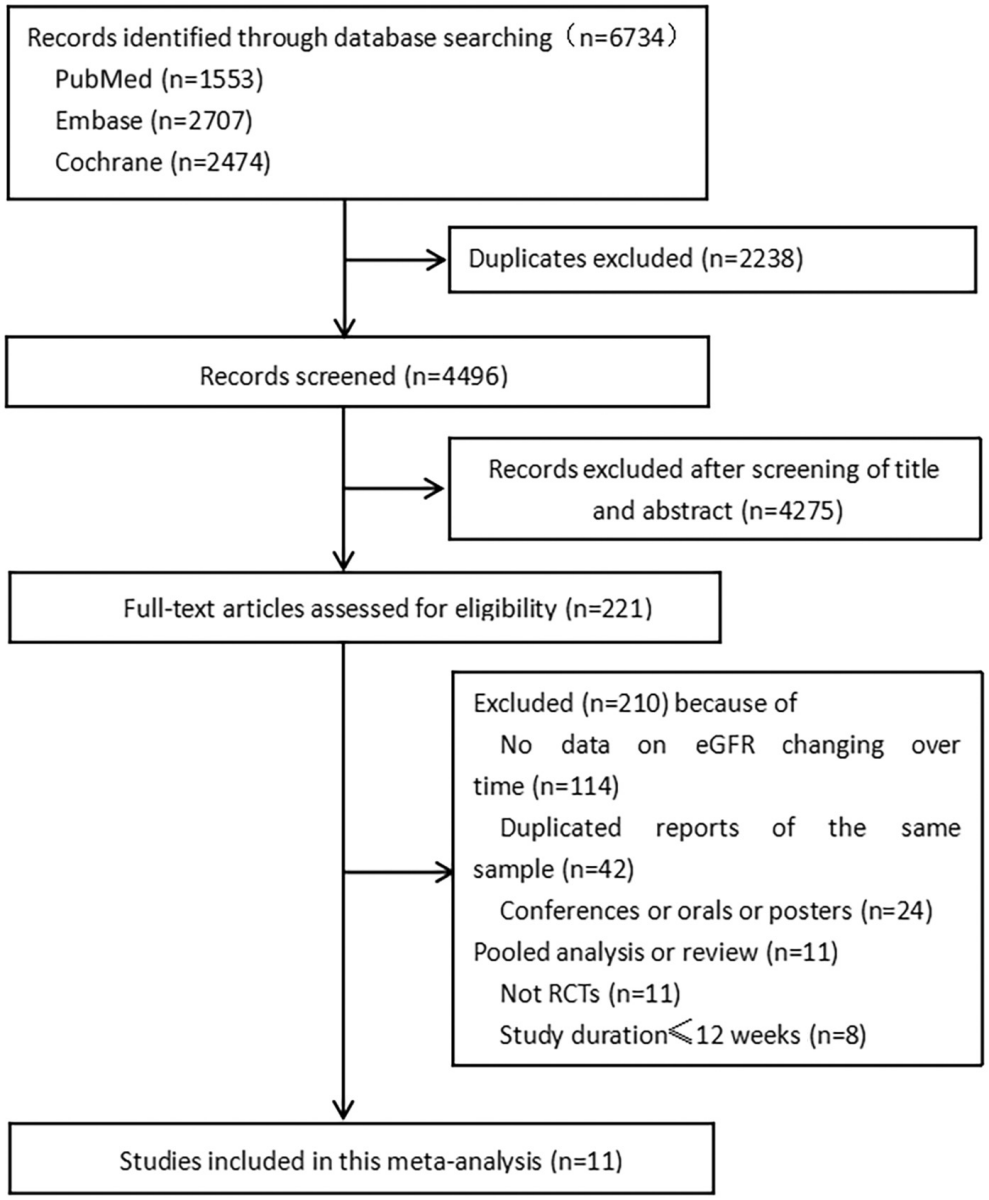
Study screening and selection process. eGFR, estimated glomerular filtration rate; RCT, randomized controlled trial

The study screening and selection process is shown in Figure 1. Of the 6734 records retrieved through the database search, 11 were included in the systematic review and meta‐analysis. The characteristics of the included studies and their participants are summarized in Table 1. The total number of participants was 58 534 (31 359 in the SGLT‐2 inhibitor group and 27 175 in the placebo group). All the included studies were double‐blinded RCTs. For the included 11 studies, two studies (Dapagliflozin Evaluation in Patients With Inadequately Controlled Type 1 Diabetes [DEPICT] and inTandem) included patients with type 1 diabetes mellitus (T1DM)^21^, ^22^; two studies (EMPEROR‐Reduced and Study to Evaluate the Effect of Dapagliflozin on the Incidence of Worsening Heart Failure or Cardiovascular Death in Patients With Chronic Heart Failure [DAPA‐HF]) included patients with heart disease with a left ventricular ejection fraction less than 40%, regardless of T2DM^11^, ^23^; one study (Study to Evaluate the Effect of Dapagliflozin on Renal Outcomes and Cardiovascular Mortality in Patients With Chronic Kidney Disease [DAPA‐CKD]) included patients with CKD with the eGFR ranging from 25 to 75 ml/min/1.73 m^2^;^24^ the remaining six studies included patients with T2DM.^6^, ^7^, ^10^, ^25^, ^26^, ^27^ Except for the inclusion criteria of the DECLARE‐TIMI58 study, which included patients with eGFR >60 ml/min/1.73 m^2^,^10^ all the other studies defined CKD as eGFR < 60 ml/min/1.73 m^2^. All included studies were conducted in multiple centers and countries. Three of the 11 studies were pooled analyses of two parallel‐group RCTs.^6^, ^21^, ^22^ The SGLT‐2 inhibitors included dapagliflozin, canagliflozin, empagliflozin, ertugliflozin, and sotagliflozin. The treatment duration ranged from 24 weeks to 4.2 years.

**TABLE 1:** The baseline characteristics of the included studies

Author	Name of study	Study design	Patient population	Location	Number of participants	Mean age (years)	Males(%)	eGFR inclusion criteria (ml/min/1.73 m^2^)	UACR inclusion criteria (mg/g)	Baseline eGFR (ml/min/1.73 m^2^)	Baseline UACR (IQR) (mg/g)	Medication and dosages	Median follow‐up (weeks)
Perkovic	CANVAS^6^	Randomized, double‐blind with placebo	Type 2 diabetes with high risk of cardiovascular events	667 centers in 30 countries	10 142	63.3 ± 8.3	64.2	>30	None	76.5 ± 20.5	22	Canagliflozin 100 mg and 300 mg	188
Perkovic	CREDENCE^27^	Randomized, double‐blind with placebo	type 2 diabetes with albuminuric CKD	695 centers in 34 countries	4401	63.0 ± 9.2	43.4	30 to <90	300 to <5000	56.8 ± 18.5	927 (463–1833)	Canagliflozin 100 mg and 300 mg	136
Heerspink	DAPA‐CKD^24^	Randomized, double‐blind with placebo	Patients with chronic kidney disease	386 centers in 21 countries	4304	51.2 ± 13.1	67.4	25 to <75	200 to <5000	43.1 ± 12.4	900 (539.6–1515.0)	Dapagliflozin 10 mg	125
Jhund	DAPA‐HF^23^	Randomized, double‐blind with placebo	Patients with HFrEF <40%)	410 centers in 20 countries	4743	66.2 ± 11.0^a^	76.2^a^	>30	None	66.0 ± 19.6^a^	None	Dapagliflozin 10 mg	102
Mosenzon	DECLARE‐TIMI58^10^	Randomized, double‐blind with placebo	Type 2 diabetes with ASCVD or multiple risk factors for ASCVD	882 centers in 33 countries	17 160	64.8 ± 5.6	63.1^a^	>60	None	64.8 ± 5.6	None	Dapagliflozin 10 mg	219
Groop	DEPICT 1 and 2^21^	Randomized, double‐blind with placebo	Type 1 diabetes	291 centers in 24 countries	251	45.6 ± 13.7^a^	52^a^	>45	>30	86.1 ± 23.7^a^	287.1 (603.1)^a^	Dapagliflozin 5 mg and 10 mg	52
Fioretto	DERIVE^26^	Randomized, double‐blind with placebo	Type 2 diabetes with moderate renal impairment	88 centers in 8 countries	321	65.3 (unclear SD)^a^	56.9^a^	45 to 59	None	53.3 ± 8.7	23.5 (2.7–5852.0)	Dapagliflozin 10 mg	24
Christoph	EMPA‐REG^7^	Randomized, double‐blind with placebo	Type 2 diabetes with established cardiovascular disease	590 centers in 42 countries	7020	63.2 ± 8.6^a^	71.5	>30	None	74.0 ± 21.4^a^	None	Empagliflozin 10 mg and 25 mg	135
Packer	EMPEROR‐Reduced^11^	Randomized, double‐blind with placebo	Patients with class II‐IV heart failure and ejection fraction <40%	520 centers in 20 countries	3730	67.2 ± 10.8^a^	76.5^a^	>20	None	61.8 ± 21.7^a^	None	Empagliflozin 10 mg	124
Cherney	VERTIS CV^25^	Randomized, double‐blind with placebo	Type 2 diabetes with established atherosclerotic cardiovascular disease	567 centers in 34 countries	8246	64.4 ± 8.1	70	>30	None	76.0 ± 20.9	19.0 (6.0–68.0)	Ertugliflozin 5 mg and 15 mg	182
Raalte	inTandem 1 and 2^22^	Randomized, double‐blind with placebo	Type 1 diabetes	171 centers in 20 countries	1575	44.0 ± 13.4^a^	48.2^a^	>45	None	97.0 ± 17.7^a^	6.3 (4.2‐12.3)^a^	Sotagliflozin 200 mg and 400 mg	52

Abbreviations: ASCVD, atherosclerotic cardiovascular disease; CANVAS, CANagliflozin cardioVascular Assessment Study; CKD, chronic kidney disease; CREDENCE, Computed TomogRaphic Evaluation of Atherosclerotic DEtermiNants of Myocardial IsChEmia; DAPA‐CKD, Study to Evaluate the Effect of Dapagliflozin on Renal Outcomes and Cardiovascular Mortality in Patients With Chronic Kidney Disease; DAPA‐HF, Study to Evaluate the Effect of Dapagliflozin on the Incidence of Worsening Heart Failure or Cardiovascular Death in Patients With Chronic Heart Failure; DECLARE‐TIMI58, Multicenter Trial to Evaluate the Effect of Dapagliflozin on the Incidence of Cardiovascular Events; DEPICT, Dapagliflozin Evaluation in Patients With Inadequately Controlled Type 1 Diabetes; DERIVE, Study to Evaluate the Effect of Dapagliflozin on Blood Glucose Level and Renal Safety in Patients With Type 2 Diabetes; eGFR, estimated glomerular filtration rate; EMPA‐REG, Empagliflozin Cardiovascular Outcome Event Trial in Type 2 Diabetes Mellitus Patients; EMPEROR‐Reduced, EMPagliflozin outcomE tRial in Patients With chrOnic heaRt Failure With Reduced Ejection Fraction; HFrEF, heart failure and reduced ejection fraction; IQR, interquartile range; NA, not available; UACR, urine albumin‐to‐creatinine ratio; VERTIS CV, Cardiovascular Outcomes Following Ertugliflozin Treatment in Type 2 Diabetes Mellitus Participants With Vascular Disease.

^a^ If the study did not show the overall characteristics of the Ssdium‐glucose cotransporter‐2 inhibitor (SGLT‐2i) group and the comparison group, we chose the baseline data described in the SGLT‐2i group.

### 3.2 Assessment of study quality and risk of bias

The Cochrane Collaboration risk of bias tool was used to assess the quality of the included trials. The risk of bias assessment is summarized in Appendix S3. Nine of the 11 studies demonstrated a low risk of bias in the areas of random sequence generation, allocation concealment, and blinding of participants and personnel.^6^, ^7^, ^10^, ^21^, ^23^, ^24^, ^25^, ^26^, ^27^ One study did not describe the method of random sequence generation,^22^ and one did not describe the method of allocation concealment.^11^ Four studies demonstrated an unclear risk of the blinding outcome assessment.^7^, ^11^, ^25^, ^26^ All studies showed low risk in selective reporting, and two reported incomplete data due to the loss of follow‐up in the long‐period medication.^6^, ^11^

### 3.3 Changes in eGFR

**FIGURE 2:**
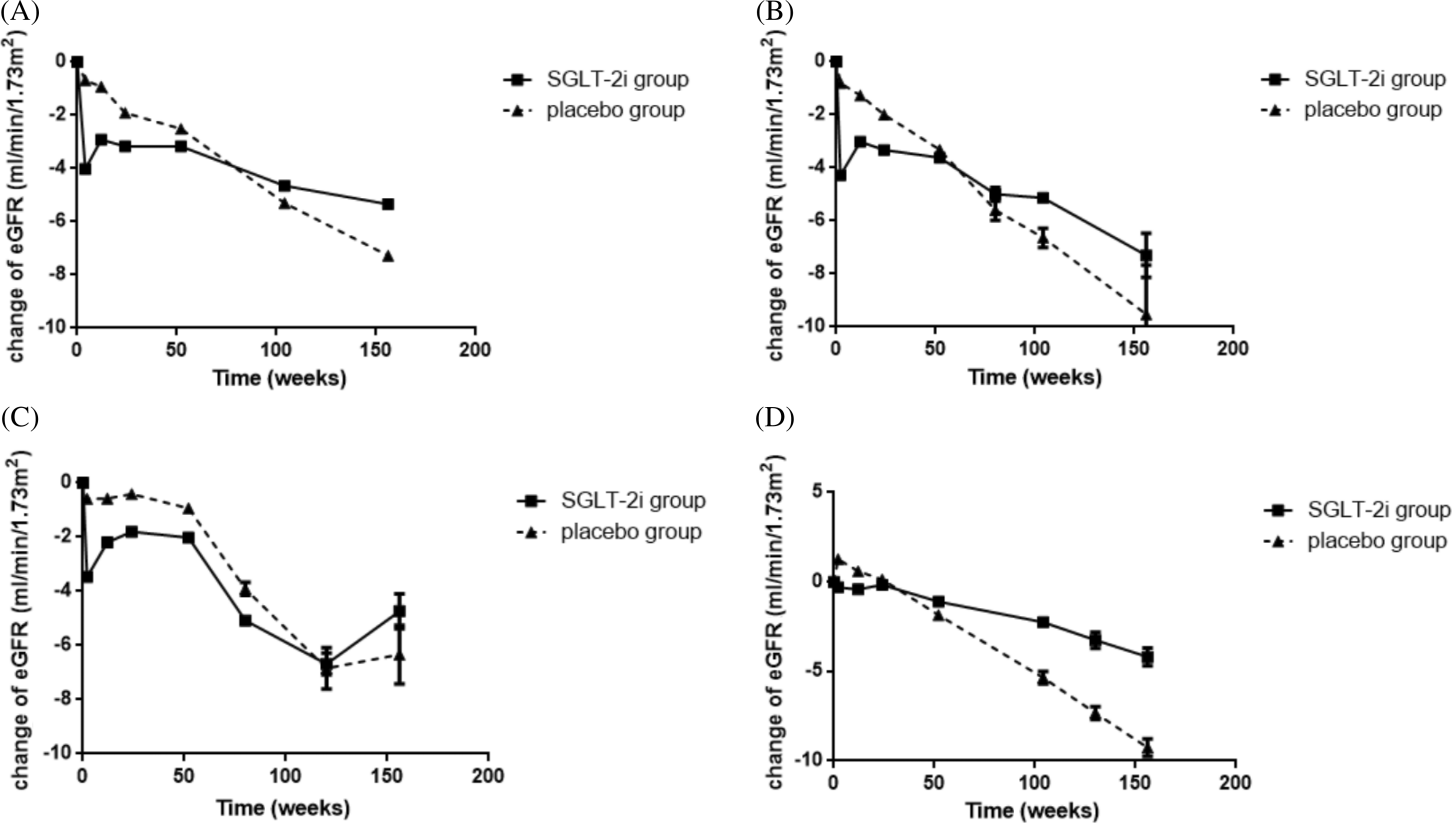
The change in eGFR during the process of administration in participants with different eGFR. (A) The change in eGFR in all participants; (B) The change in eGFR in subgroup of eGFR > 60 ml/min/1.73 m^2^; (C) The change in eGFR in the subgroup of eGFR<60 ml/min/1.73 m^2^; (D) The change in eGFR in the subgroup of eGFR < 30 ml/min/1.73 m^2^. *In (C) (eGFR < 60 ml/min/1.73 m^2^), only two studies (DAPA‐CKD, DECLARE‐TIMI 58) were followed up for more than 150 weeks with relatively wide confidence interval (95% CI −2.06 to 11.54). eGFR, estimated glomerular filtration rate; SGLT‐2i, sodium/glucose cotransporter‐2 inhibitors

The changes in eGFR during the whole process of administration are shown in Figure 2A and Appendix S4. There was no heterogeneity in the baseline eGFR in the included 11 studies (*I* ^2^ = 0%, *p* = .95). In the first 2–4 weeks, there was an acute decline of eGFR in the SGLT‐2i group compared with the placebo group, with an WMD of −3.36 ml/min/1.73 m^2^ (8 studies, 95% CI, −3.66 to −3.06; *I* ^2^ = 35%, *p* = .15); when compared with baseline eGFR in the SGLT‐2i group, the WMD was −4.02 ml/min/1.73 m^2^ (95% CI, −4.44 to −3.61; *I* ^2^ = 0%, *p* = .45). Afterwards, the decline rate of eGFR gradually slowed down in the SGLT‐2i group, the WMD compared with the controls in the 12th–18th weeks, 24th–32th weeks and 52th week (1 year) was −1.93 ml/min/1.73 m^2^ (10 studies, 95% CI, −2.18 to −1.68; *I* ^2^ = 35%, *p* = .13), −1.36 ml/min/1.73 m^2^ (10 studies, 95% CI, −1.64 to −1.08; *I* ^2^ = 0%, *p* = .61), and − 0.81 ml/min/1.73 m^2^ (10 studies, 95% CI, −1.06 to −0.56; *I* ^2^ = 0%, *p* = .68), respectively. Figure 2A demonstrated that the decline in eGFR was lower in the SGLT‐2i group at less than 100 weeks and remained stable. By the 104th week, changes in eGFR from the baseline were similar between the SGLT‐2i (−4.65 ml/min/1.73 m^2^) and placebo groups (−5.30 ml/min/1.73 m^2^), with a WMD of 0.35 ml/min/1.73 m^2^ (eight studies, 95% CI, 0.04 to 0.66; *I* ^2^ = 45%, *p* = .08). During the next few years, SGLT‐2i persistently slowed down the decline of eGFR, the WMD of eGFR between the two groups was 1.32 ml/min/1.73 m^2^ (six studies, 95% CI, 0.97 to 1.67; *I* ^2^ = 33%, *p* = .19), 1.77 ml/min/1.73 m^2^ (four studies, 95% CI, 1.35 to 2.19; *I* ^2^ = 40%, *p* = .17) and 2.55 ml/min/1.73 m^2^ (two studies, 95% CI, 1.58 to 3.53; *I* ^2^ = 0%, *p* = .32) at the 156th week(the third year), 208th week (the fourth year), 260th week (the fifth year), respectively.

**FIGURE 3:**
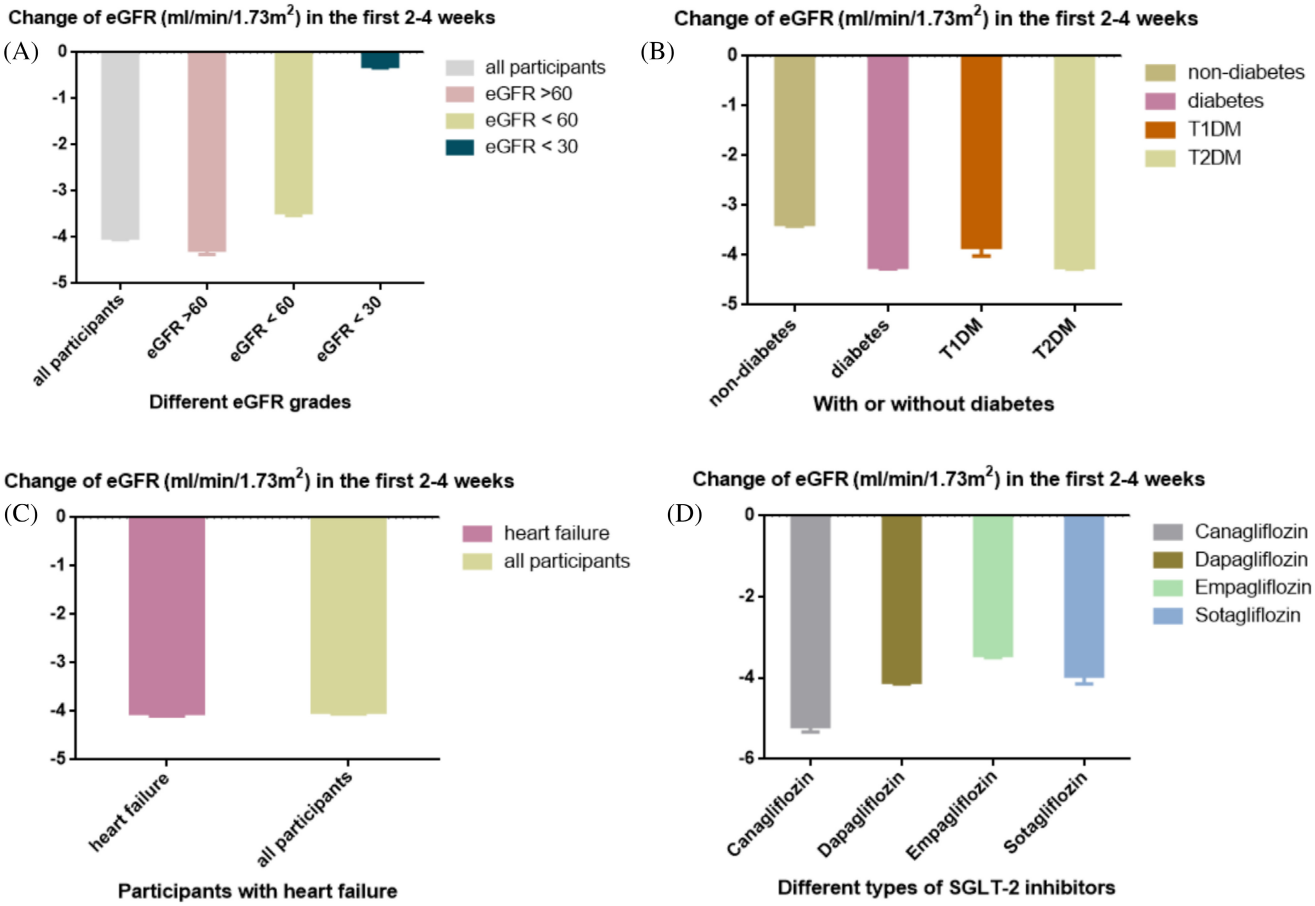
The rapid decline in eGFR in the first 2–4 weeks of administration. (A) The decline in eGFR in different eGFR grades; (B) The decline in eGFR in participants with or without diabetes and different types of diabetes; (C) The decline in eGFR in participants with heart failure and in all participants; (D) The decline in eGFR in participants with different types of SGLT‐2i. CI, confidence interval; eGFR, estimated glomerular filtration rate; SGLT‐2i, sodium‐glucose cotransporter‐2 inhibitors; T1DM, type 1 diabetes mellitus; T2DM, type 2 diabetes mellitus

Because a rapid decline in eGFR in the first 2–4 weeks of administration was observed in the SGLT‐2i group, a further analysis was performed to explore the factors associated with the degree of eGFR decline. All participants were divided by different eGFR grades, with or without diabetes, types of diabetes, with or without heart failure, and different types of SGLT2‐i **(**Figure 3 **)**. Figure 3A demonstrated that participants with eGFR < 30 ml/min/1.73 m^2^ showed only a slight decline in eGFR in the initial administration (0.3 ml/min/1.73 m^2^), whereas a rapid decline in eGFR was observed in other eGFR grades. Participants with diabetes showed a greater decline in eGFR compared with participants without diabetes (4.24 ml/min/1.73 m^2^ vs 3.38 ml/min/1.73 m^2^) (Figure 3B), and the decline in eGFR was similar between T1DM and T2DM. For patients with heart failure, the decline in eGFR was similar to that in all participants (4.04 ml/min/1.73 m^2^ versus 4.02 ml/min/1.73 m^2^) **(**Figure 3C **)**. However, the degree of eGFR decline varied slightly across different SGLT‐2i, with the least in the empagliflozin group (3.44 ml/min/1.73 m^2^) and the most in the canagloflozin group (5.20 ml/min/1.73 m^2^) **(**Figure 3D **)**.

#### 3.3.1 Subgroup analysis in the SGLT‐2i group

##### Patients with different baseline eGFR

A subgroup analysis according to different baseline eGFR was performed to explore the changes in eGFR in different CKD grades. However, only a few RCTs reported the change differed by different eGFR. Four of the 11 RCTs (DAPA‐CKD, DAPA‐HF, DECLARE‐TIM 58, EMPEROR‐Reduced) reported changes in renal function in patients with eGFR > 60 ml/min/1.73 m^2^ and those with eGFR < 60 ml/min/1.73 m^2^, separately^10^, ^23^, ^28^, ^29^; and three of the RCTs (CREDENCE, DAPA‐CKD, EMPA‐REG) reported changes in renal function in patients with eGFR < 30 ml/min/1.73 m^2^.^28^, ^30^, ^31^ As shown in Figure 2B (eGFR > 60 ml/min/1.73 m^2^) and Figure 2C (eGFR <60 ml/min/1.73 m^2^), there was a rapid decline in eGFR during the initial administration, but the decline quickly reversed and the decline rate of eGFR was slower compared to the placebo group. In Figure 2C (eGFR < 60 ml/min/1.73 m^2^), only two studies (DAPA‐CKD, DECLARE‐TIMI58) were followed up for more than 150 weeks with relatively wide CI (95% CI −2.06 to 11.54), so the improvement at approximately 150 weeks did not reflect the actual change in eGFR. In the subgroup with baseline eGFR < 30 ml/min/1.73 m^2^ (Figure 2D), there was no remarkable decline in eGFR during the initial stage of administration, and eGFR was consistently higher in the SGLT‐2i group before the 50th week. Compared with placebo, SGLT‐2i could effectively slow down the decline in eGFR regardless of baseline renal function.

##### Patients with or without diabetes

**FIGURE 4:**
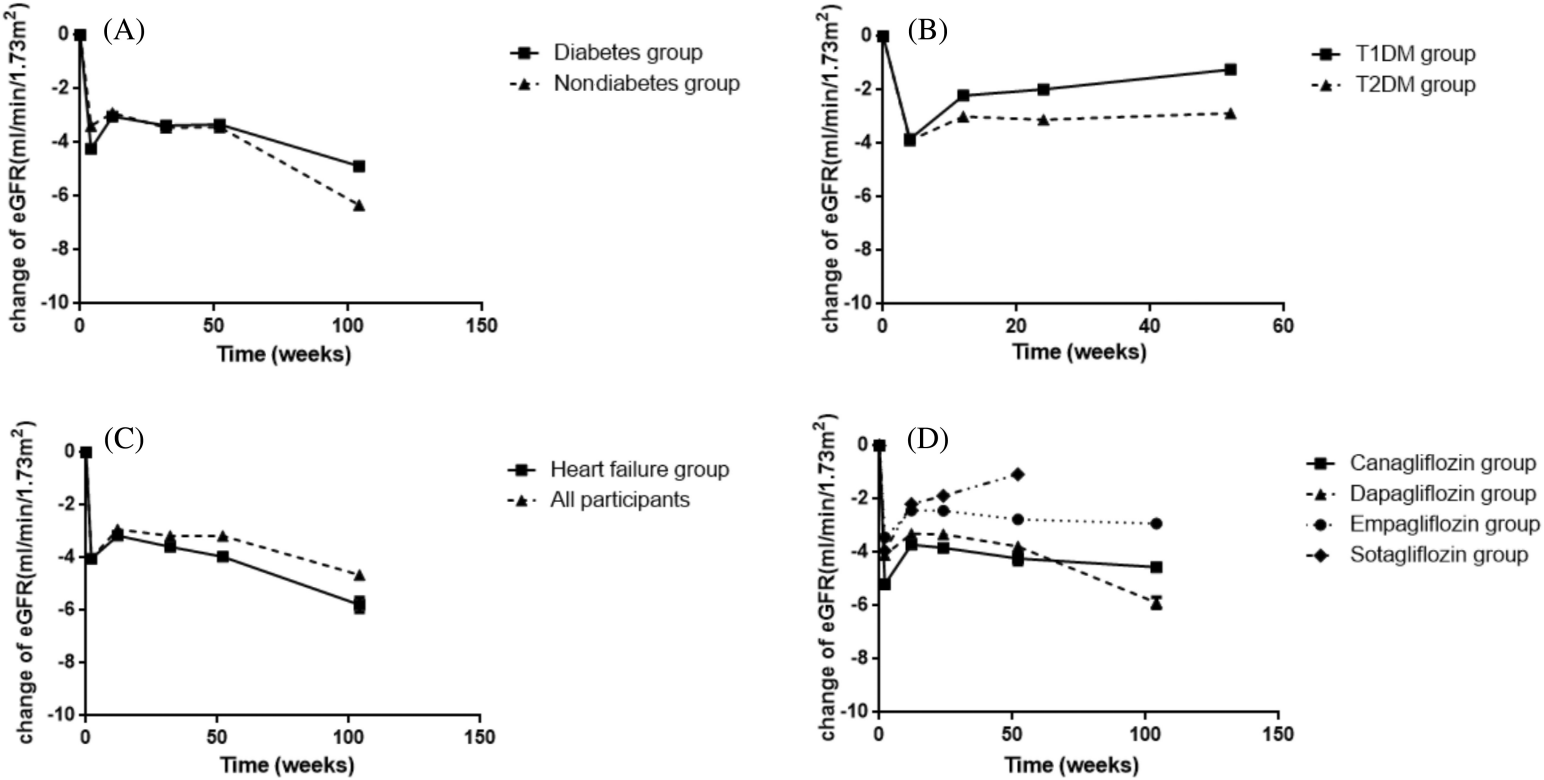
The change in eGFR during the process of administration in participants with different underlying disease and different types of SGLT‐2i. (A) The change in eGFR in diabetic or nondiabetic participants; (B) The change in eGFR in participants with T1DM or T2DM; (C) The change in eGFR in participants with heart failure and in all participants; (D) The change in eGFR in participants with different types of SGLT‐2i. eGFR, estimated glomerular filtration rate; SGLT‐2i, sodium‐glucose cotransporter‐2 inhibitors; T1DM, type 1 diabetes mellitus; T2DM, type 2 diabetes mellitus

Three of the included RCTs involved participants who did not have diabetes (DAPA‐CKD, DAPA‐HF, EMPEROR‐Reduced). We compared the effect of SGLT‐2i on eGFR in the diabetic and nondiabetic groups and found that the changes were similar in the two subgroups (Figure 4A). The eGFR decreased rapidly in the initial administration in both groups and then followed by stabilization. In the nondiabetic group, the initial decline of eGFR was lower than in the diabetic group. In addition, we also compared the changes in eGFR in patients with T1DM and those with T2DM. As shown in Figure 4B, the change trend of eGFR was similar in the two groups. However, the two studies included T1DM participants(inTandem and DEPICT) were followed for only 52 weeks, and we cannot draw longer conclusions.

##### Patients with or without heart failure

Two of the included studies (DAPA‐HF and EMPEROR‐Reduced) enrolled participants with heart failure (ejection fraction <40%). Figure 4C showed the changes in eGFR (SGLT‐2i group) in patients with heart failure compared with all participants. During the follow‐up of 104 weeks, there was a rapid decline of eGFR in the heart failure subgroup, and then the change in eGFR gradually stabilized, which was similar to that in all participants.

##### Patients with different types of SGLT‐2i

All included studies were grouped according to different types of SGLT‐2i. Participants were treated with canagliflozin in two studies (CANVAS and CREDENCE), dapagliflozin in five studies (DAPA‐CKD, DAPA‐HF, DECLARE‐TIMI58, DEPICT, and A Study to Evaluate the Effect of Dapagliflozin on Blood Glucose Level and Renal Safety in Patients With Type 2 Diabetes [DERIVE]), empagliflozin in two studies (EMPA‐REG and EMPEROR‐Reduced), and sotagliflozin in one study (inTandem 1 and 2). As shown in Figure 4D, the changes in eGFR were generally similar among the groups; the lowest reduction of eGFR in the initial administration was observed in the empagliflozin group, and the highest in the canagliflozin group. After the acute decline of renal function, eGFR in canagliflozin, empagliflozin and sotagliflozin group could be maintained for a long time, but there was a trend of sustaining decline in eGFR in the dapagliflozin group. Because only one study of sotagliflozin was included, the change of eGFR over a longer period was not available.

### 3.4 Assessment of funnel plot asymmetry

For the baseline eGFR in the included 11 studies, the funnel plot was roughly symmetrical on the left and right sides except for one study (DEPICT study) **(**Appendix S4 **)**. Because the sample size of the DEPICT study was relatively small (251 participants) compared with other included studies and the standard error of the study was relatively large, this study was located at the bottom of the funnel plot.

## 4 DISCUSSION

This systematic review and meta‐analysis found that SGLT‐2i could slow down the deterioration of eGFR, but there was a significant decline of eGFR in the first 2–4 weeks of administration, with 4.02 ml/min/1.73 m^2^ (95% CI, −4.44 to −3.61) compared with baseline eGFR. Subsequently, the decline rate of eGFR was slower than that of the placebo group, which suggested that the protective mechanisms of SGLT‐2i on renal function had gradually revealed; statistically significant benefit of SGLT‐2i did not appear until the 104th week of therapy. The renoprotective effect remained stable and gradually increased over the next few years. The results of subgroup analyses were generally consistent.

SGLT‐2i may have beneficial effects on kidney function through several mechanisms, including decreasing glomerular hyperfiltration, reducing sodium reabsorption in the proximal tubule, decreasing inflammatory and fibrotic responses of the kidney, and reducing UACR, which could slow the progression of diabetic nephropathy.^4^, ^32^, ^33^ Moreover, SGLT‐2i lowers blood pressure and body weight and reduces uric acid levels, which also show benefits for the cardiovascular system.^34^ Several RCTs have confirmed the long‐term benefits of SGLT‐2i use on kidney function. The CREDENCE study found a 32% lower relative risk of end‐stage kidney disease in the canagliflozin group than in the placebo group, and the relative risk of renal‐specific composite end point (end‐stage kidney disease, a doubling of the creatinine level, or death from renal causes) was reduced by 34%.^27^ The EMPA‐REG study found a 55% lower relative risk of renal‐replacement therapy in the empagliflozin group compared with placebo.^7^ The DAPA‐CKD study also found that dapagliflozin could decrease the risk for the composite renal outcome of a sustained decline in eGFR of at least 50%.^24^ These RCTs included patients with eGFR >30 ml/min/1.73 m^2^, which indicated the efficacy and safety of SGLT‐2i in patients with renal impairment. Moreover, the DAPA‐CKD study also included patients with CKD without T2DM, and a subgroup analysis found that the effects of dapagliflozin were similar in patients without T2DM.^24^ All the RCTs demonstrated an acute decline of eGFR when SGLT‐2i was initially used, and the renal function recovered subsequently because of the renoprotective mechanism of SGLT‐2i. However, no meta‐analysis focused on the early decline and persistent change in eGFR. Our meta‐analysis evaluated the results of 11 RCTs and discovered an obvious decline in eGFR during the first 2–4 weeks; afterwards the renoprotective mechanism of SGLT‐2i gradually appeared and remained persistently, but statistically significant benefit of SGLT‐2i did not appear until the 104th week after continuous medication.

The macula densa in the proximal tubule regulates the contraction and dilatation of the afferent arterioles in keeping with the sodium concentration and renal plasma flow, which is called the tubuloglomerular feedback mechanism. The increase in renal plasma flow leads to an increased sodium concentration in the macula densa, angiotensin II is produced, and the afferent arteriolar contraction reduces blood flow into the glomerulus. In contrast, when the renal plasma flow and sodium concentration in the macula densa decreases, the angiotensin II level declines, and the afferent arteriole dilates.^35^ The tubuloglomerular feedback maintains the relative stability of the GFR. SGLT‐2i increases the glucose and sodium delivery in the proximal tubule, leading to afferent arteriolar vasoconstriction, thereby suppressing glomerular hyperfiltration.^36^ However, owing to its influence on renal hemodynamic function, a transient decline in renal function is usually observed during the early stage when SGLT‐2is are used. Therefore, it is necessary to know the duration and extent of the decline in eGFR when SGLT‐2i is used, which may be relatively acceptable and safe for the kidney.

The DIAMOND study examined the renal effects after SGLT‐2i discontinuation in patients diagnosed with CKD.^37^ In the study, each participant received dapagliflozin 10 mg daily for 6 weeks and a 6‐week washout period with placebo in between. The measured GFR (mGFR) declined by −6.6 ml/min per 1.73 m^2^ at the sixth week of dapagliflozin treatment, but the reduction was fully reversible within 6 weeks after withdrawal. The findings in the DIAMOND study suggested that the acute decrease in mGFR was due to the natriuresis caused by SGLT‐2i, leading to systemic and intrarenal hemodynamic effects. The decline of eGFR in DIAMOND study was consistent with the eGFR change in the initial administration in this meta‐analysis, suggesting that the initial decline of eGFR was probably because of the diuretic effect of SGLT‐2i, and that the renal protective effect of SGLT‐2i may have been exerted since the initial administration.

To our knowledge, this is the first meta‐analysis and systematic analysis to investigate the influence of SGLT‐2i on eGFR during the course of administration. Our detailed search strategy spanned several databases to retrieve all eligible studies. All the RCTs were conducted at multiple centers in different countries, with a follow‐up period of more than 1 year (except for the DERIVE study), and there was no heterogeneity of baseline eGFR among the included studies, demonstrating the high quality and consistency of our meta‐analysis. Another strength of our study was that we did not exclude patients with CKD or nondiabetics. This showed the safety of SGLT‐2i in patients with renal impairment and indicated that SGLT‐2i could slow the progression of renal insufficiency even in patients without diabetes. The subgroup analysis also confirmed the benefits of SGLT‐2i in patients with different baseline eGFRs. Although a previous meta‐analysis^38^ planned to explore the effect of SGLT‐2i on renal function, they excluded three studies of populations with renal impairment when they performed a pooled analysis and found no significant change in eGFR between the SGLT‐2i and placebo groups. Another meta‐analysis^39^ found that SGLT‐2i significantly slowed the decline in eGFR in patients with a treatment duration of >52 weeks compared with placebo, but they did not compare the early change in eGFR between the two groups. Furthermore, two RCTs (Cardiovascular Outcomes Following Ertugliflozin Treatment in Type 2 Diabetes Mellitus Participants With Vascular Disease [VERTIS CV] and inTandem)^22^, ^25^ in our study included SGLT‐2i, which had not yet been introduced to the market (ertugliflozin and sotagliflozin). The conclusions from these two RCTs were consistent with those of the other studies, and the results of our meta‐analysis remained unchanged after excluding them.

Our study had some limitations. First, because the results of several studies were presented only as figures in the respective articles and were not available as raw data, we used image extraction software to extract data (WebPlotDigitizer, version 4.5), which may have led to inaccurate results. However, all the results of eGFR in the pooled analysis at every time point were not heterogeneous; hence we believe that the results should be credible. Second, only a few of the included RCTs analyzed the data of CKD patients separately, and the results of subgroup analysis may be less convincing. Third, further classification of renal function was not available (eGFR range from 30‐‐44 ml/min/1.73 m^2^ and 45‐59 ml/min/1.73 m^2^, respectively) in the included studies, and it would be an opportunity for future research. Fourth, the kidney outcomes we explored in this study were not primary outcomes in most included studies. Fifth, there was a loss of follow‐up data in several RCTs owing to the long follow‐up period, suggesting the possibility of attrition bias.

## 5 CONCLUSION

In conclusion, SGLT‐2 inhibitors could slow down the deterioration of renal function, regardless of the CKD grades and diabetes, but there was an acute decline of eGFR in the first 2–4 weeks after administration, with a reduction of 4.02 ml/min/1.73 m^2^ (95% CI, −4.44 to −3.61) compared with baseline eGFR; then the renoprotective effect of SGLT‐2i gradually appeared. After the first 2–4 weeks, the decline rate of eGFR in the SGLT‐2i group was always slower than that of the placebo group, and statistically significant benefit of SGLT‐2i appeared at about the 104th week of administration, after which the renoprotective effect of SGLT‐2i remained stable and enhanced with time. These data provide an appropriate range of decline in eGFR during the first 2 to 4 weeks—a decline in eGFR within about 4 ml/min/1.73 m^2^ may be generally considered safe. If the eGFR decreases by more than 4 ml/min/1.73 m^2^, the administration of SGLT‐2 should be done with caution; but the decline of eGFR is mostly reversible after drug withdrawal. This meta‐analysis provides substantive evidence supporting the benefits of SGLT‐2 inhibitors in slowing down the deterioration of renal function and the safety of its initial administration, even in patients with renal impairment or without diabetes.

## AUTHOR CONTRIBUTIONS

Weigang Zhao conceived the systematic review and contributed to the planning, methodology, drafting, and revision of the manuscript. Yanbei Duo and Junxiang Gao performed systematic searches, study selection, and data extraction and analysis. Yanbei Duo wrote the first draft, with adjudications made by Weigang Zhao and Tao Yuan. All the authors contributed to the writing and revision of the manuscript. All the authors approved the final draft of the manuscript.

## FUNDING INFORMATION

The authors have no funding sources available for this study. All authors had full access to all data in the study and agreed to the decision to submit it for publication.

## CONFLICT OF INTEREST

The authors declare that they have no conflict of interest.

## ETHICAL STATEMENT

No human or animal studies involved for the study.

## Supporting information


**Appendix S1.** The prespecified protocol of this meta‐analysis.

**Appendix S2.** Preferred Reporting Items for Systematic Reviews and Meta‐Analysis (PRISMA) statement.

**Appendix S3.** Study quality and risk of bias assessment.

**Appendix S4.** The changes in eGFR during the process of administration and the funnel plot of included eleven studies.
