# Supplementary material for: Effect of sodium‐glucose cotransporter 2 inhibitors on the rate of decline in kidney function: A systematic review and meta‐analysis
Source: J Diabetes. 2023 Jan 6;15(1):58–70. doi: 10.1111/1753-0407.13348 (PMC9870734; doi:10.1111/1753-0407.13348)
Supplement: Supplementary file 1 — Appendix S1. The prespecified protocol of this meta‐analysis. [file JDB-15-58-s001.docx]

**Supplementary Appendix 1**

1. Title: Effect of Sodium-Glucose Cotransporter 2 inhibitors on the rate of decline in kidney function: A systematic review and meta-analysis
2. **Objectives:** This study aimed to investigate the effects of sodium-glucose cotransporter 2 (SGLT2) inhibitors on renal outcomes in the initial medication and long period of follow-up.
3. **Protocol and registration:** Methods of database search, study selection, data extraction, assessment of study quality and risk of bias, and statistical analysis were prespecified in the protocol at the beginning of the study.
4. **Reporting:** This systematic review and meta-analysis was reported according to the Preferred Reporting Items for Systematic Reviews and Meta-Analyses (PRISMA) statement.
5. **Eligibility criteria**
   1. Study characteristics
      1. Population: patients who use SGLT-2 inhibitors
      2. Intervention: SGLT2 inhibitors
      3. Comparison: placebo or other antidiabetic drugs
      4. Outcomes of interests
         1. decline of estimated glomerular filtration rate (eGFR) in the initial medication of SGLT-2 inhibitors
         2. Changes in estimated glomerular filtration rate (eGFR) in the long period of mediation of SGLT-2 inhibitors
         3. Changes in urine albumin-to-creatinine ratio (UACR)
      5. Study design: randomized controlled trials (RCTs)
      6. Length of follow-up: at least 12 weeks of study duration
   2. Report characteristics
      1. Years considered: published until Aug 31, 2021
      2. Language: English published only
      3. Publication status: full-text articles with no limitation for publication status
   3. Inclusion and exclusion criteria
      1. The study population comprised men and women who use SGLT-2 inhibitors.
      2. We included RCTs comparing the efficacy or safety of SGLT2 inhibitors with that of placebo or other antidiabetic drugs, regardless of the presence of diabetes in patients and the baseline eGFR.
      3. Any concurrent use of antidiabetic drugs was allowed including oral antidiabetic drugs, insulin, and glucagon-like peptide-1 receptor agonists other than those for intervention and comparison.
      4. Only RCTs with a study duration of ≥ 12 weeks were included.
      5. We only included RCTs reporting at least the changes of eGFR through the whole medication.
      6. In case of duplicates or extensions, we only included the study with the longer duration or more information about renal outcomes.
      7. Pooled analyses or secondary analyses were only included only when they provided more information about renal outcomes than original articles.
      8. We included full-text articles with no limitation for publication status.
6. **Information sources:** We searched the electronic databases including MEDLINE, Embase, and the Cochrane Central Register of Controlled Trials.
7. **Search strategy:** RCTs of SGLT2 inhibitors in patients with type 2 diabetes were searched using the following search terms:

1.MEDLINE:

Search Query Items to found

1# Sodium-Glucose Transporter 2 Inhibitors[Title/Abstract] 152

2# Sodium Glucose Transporter 2 Inhibitors[Title/Abstract] 152

3# Sodium-Glucose Transporter 2 Inhibitor[Title/Abstract] 37

4# Sodium Glucose Transporter 2 Inhibitor[Title/Abstract] 37

5# SGLT-2 Inhibitors[Title/Abstract] 714

6# SGLT 2 Inhibitors[Title/Abstract] 714

7# Gliflozins[Title/Abstract] 102

8# SGLT2 Inhibitors[Title/Abstract] 2233

9# Gliflozin[Title/Abstract] 42

10# SGLT-2 Inhibitor[Title/Abstract] 321

11# SGLT 2 Inhibitor[Title/Abstract] 321

12# SGLT2 Inhibitor[Title/Abstract] 1648

13# Inhibitor, SGLT2[Title/Abstract] 225

14# SGLT2[Title/Abstract] 4399

15# SGLT-2[Title/Abstract] 1008

16# canagliflozin[Title/Abstract] 1243

17# dapagliflozin[Title/Abstract] 1590

18# empagliflozin[Title/Abstract] 1631

19# ertugliflozin[Title/Abstract] 162

20# ipragliflozin[Title/Abstract] 240

21# Luseogliflozin[Title/Abstract] 109

22# Sotagliflozin[Title/Abstract] 101

23# Remogliflozin[Title/Abstract] 34

24# Sergliflozin[Title/Abstract] 15

25# Tofogliflozin[Title/Abstract] 124

26# OR #1-#25 6408

27# Randomized Controlled Trial[Publication Type] 538922

28# Random*[Title/Abstract] 1240244

29# RCT[Title/Abstract] 27004

30# RCTs[Title/Abstract] 39828

31# #27 OR #28 OR #29 OR #30 1374306

32# #26 AND #31 1553

2. Embase:

1# 'sodium glucose cotransporter 2 inhibitor':ab,ti 681

2# 'sodium glucose cotransporter 2 inhibitors':ab,ti 1137

3# 'sodium glucose cotransporter 2':ab,ti 3036

4# 'sglt-2 inhibitor':ab,ti 551

5# 'sglt-2 inhibitors':ab,ti 1075

6# 'sglt 2':ab,ti 1580

7# atigliflozin:ab,ti 0

8# bexagliflozin:ab,ti 9

9# canagliflozin:ab,ti 2086

10# dapagliflozin:ab,ti 2885

11# empagliflozin:ab,ti 2788

12# ertugliflozin:ab,ti 251

13# ipragliflozin:ab,ti 370

14# luseogliflozin:ab,ti 202

15# sotagliflozin:ab,ti 162

16# remogliflozin:ab,ti 62

17# sergliflozin:ab,ti 16

18# tofogliflozin:ab,ti 205

19# gliflozin:ab,ti 68

20# OR #1-#19 9374

21# random*:ab,ti 1679972

22# rct:ab,ti 42996

23# rcts:ab,ti 56043

24# #21 OR #22 OR #23 1694302

25# #20 AND #25 2707

3.The Cochrane Central Register of Controlled Trials:

1# 'sodium glucose cotransporter 2 inhibitor':ab,ti,kw 963

2# 'sodium glucose cotransporter 2 inhibitors':ab,ti,kw 677

3# 'sodium glucose cotransporter 2':ab,ti 1264

4# 'sglt-2 inhibitor':ab,ti 209

5# 'sglt-2 inhibitors':ab,ti 158

6# 'sglt 2':ab,ti 351

7# atigliflozin:ab,ti 1

8# bexagliflozin:ab,ti 22

9# canagliflozin:ab,ti 611

10# dapagliflozin:ab,ti 1274

11# empagliflozin:ab,ti 1135

12# ertugliflozin:ab,ti 149

13# ipragliflozin:ab,ti 160

14# luseogliflozin:ab,ti 85

15# sotagliflozin:ab,ti 107

16# remogliflozin:ab,ti 28

17# sergliflozin:ab,ti 5

18# tofogliflozin:ab,ti 86

19# gliflozin:ab,ti 8

20# OR #1-#19 3644

21# randomized controlled trial:pt 514388

21# random*:ab,ti 1251581

22# rct:ab,ti 37042

23# rcts:ab,ti 17304

24# #21 OR #22 OR #23 1181713

25# #20 AND #25 2474

1. **Study selection:** All identified records were screened and evaluated for eligibility by two reviewers independently. We reviewed titles, abstracts, and full texts of the studies. Any disagreements were resolved by consensus among the investigators of this study.
2. **Data extraction:** Standardized data extraction was performed by two reviewers independently as follows. Any discrepancies were resolved by consensus among the investigators of this study.
   1. First author
   2. Publication year
   3. Study Design
   4. Participants Population and location
   5. Numbers of participants
   6. Mean age of participants
   7. The proportion of males
   8. eGFR inclusion criteria(mL/min/1.73 m^2^ )
   9. UACR inclusion criteria(mg/g)
   10. Baseline eGFR(mL/min/1.73 m^2^ ) and UACR(mg/g)
   11. Intervention including names and doses of SGLT2 inhibitors
   12. Comparison including placebo or names and doses of other antidiabetic drugs
   13. Background antidiabetic drugs
   14. the changes of eGFR in the initial and long period of medication in mL/min/1.73 m^2^
   15. the changes of UACR in the long period of medication in mg/g
   16. Duration of medication and follow-up time
