# Supplementary figures and images for: Effect of sodium‐glucose cotransporter 2 inhibitors on the rate of decline in kidney function: A systematic review and meta‐analysis
Source: J Diabetes. 2023 Jan 6;15(1):58–70. doi: 10.1111/1753-0407.13348 (PMC9870734; doi:10.1111/1753-0407.13348)

**Supplementary Appendix 3**. Study quality and risk of bias assessment.


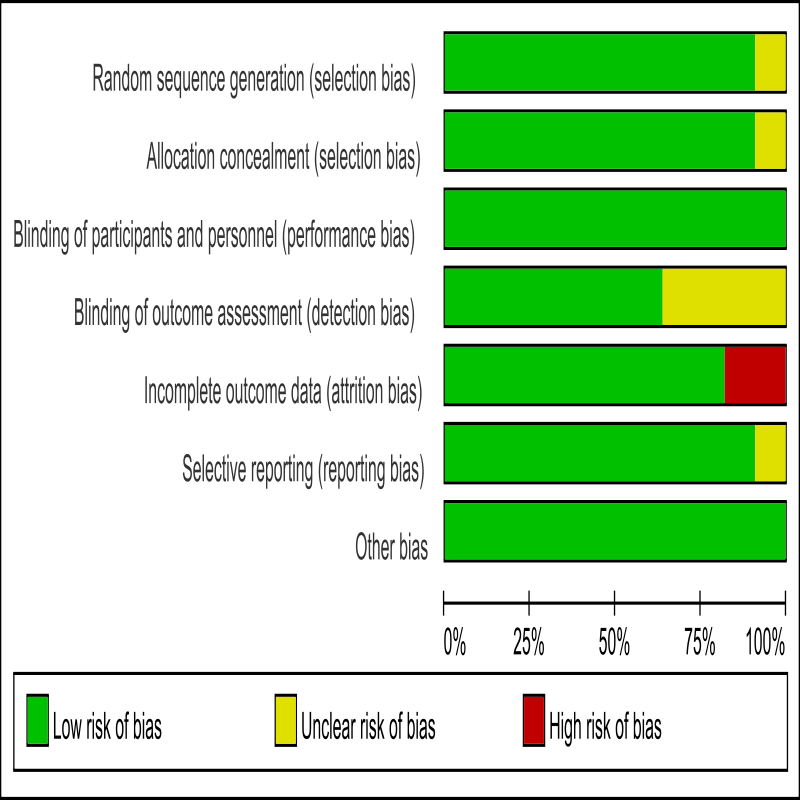


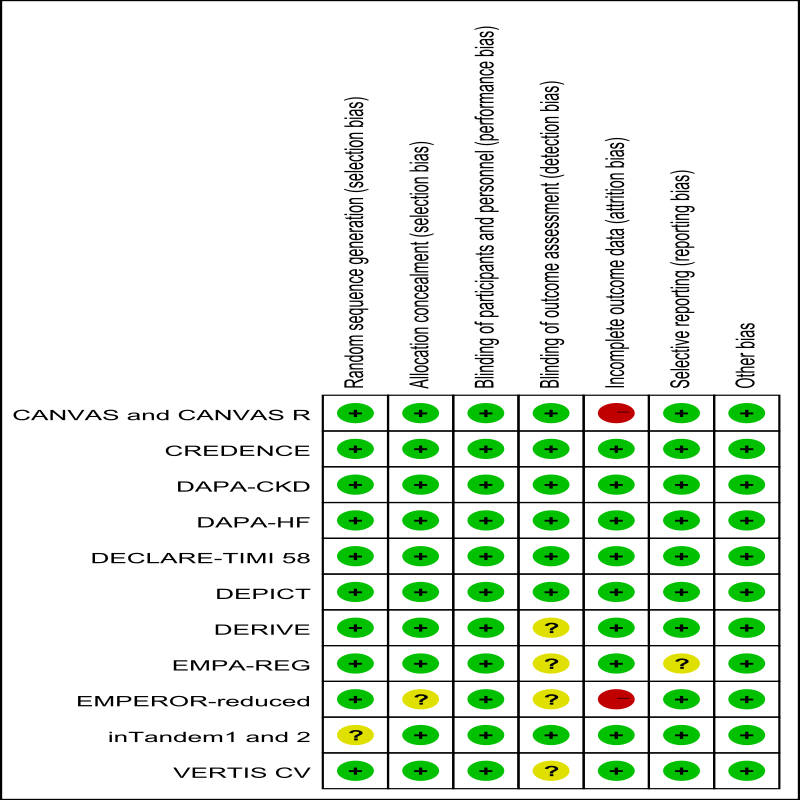

Supplement: Supplementary file 3 — Appendix S3. Study quality and risk of bias assessment. [file JDB-15-58-s004.docx]
