# Supplementary material for: Effect of sodium‐glucose cotransporter 2 inhibitors on the rate of decline in kidney function: A systematic review and meta‐analysis
Source: J Diabetes. 2023 Jan 6;15(1):58–70. doi: 10.1111/1753-0407.13348 (PMC9870734; doi:10.1111/1753-0407.13348)
Supplement: Supplementary file 4 — Appendix S4. The changes in eGFR during the process of administration and the funnel plot of included eleven studies. [file JDB-15-58-s002.docx]

**Supplementary Appendix 4**.


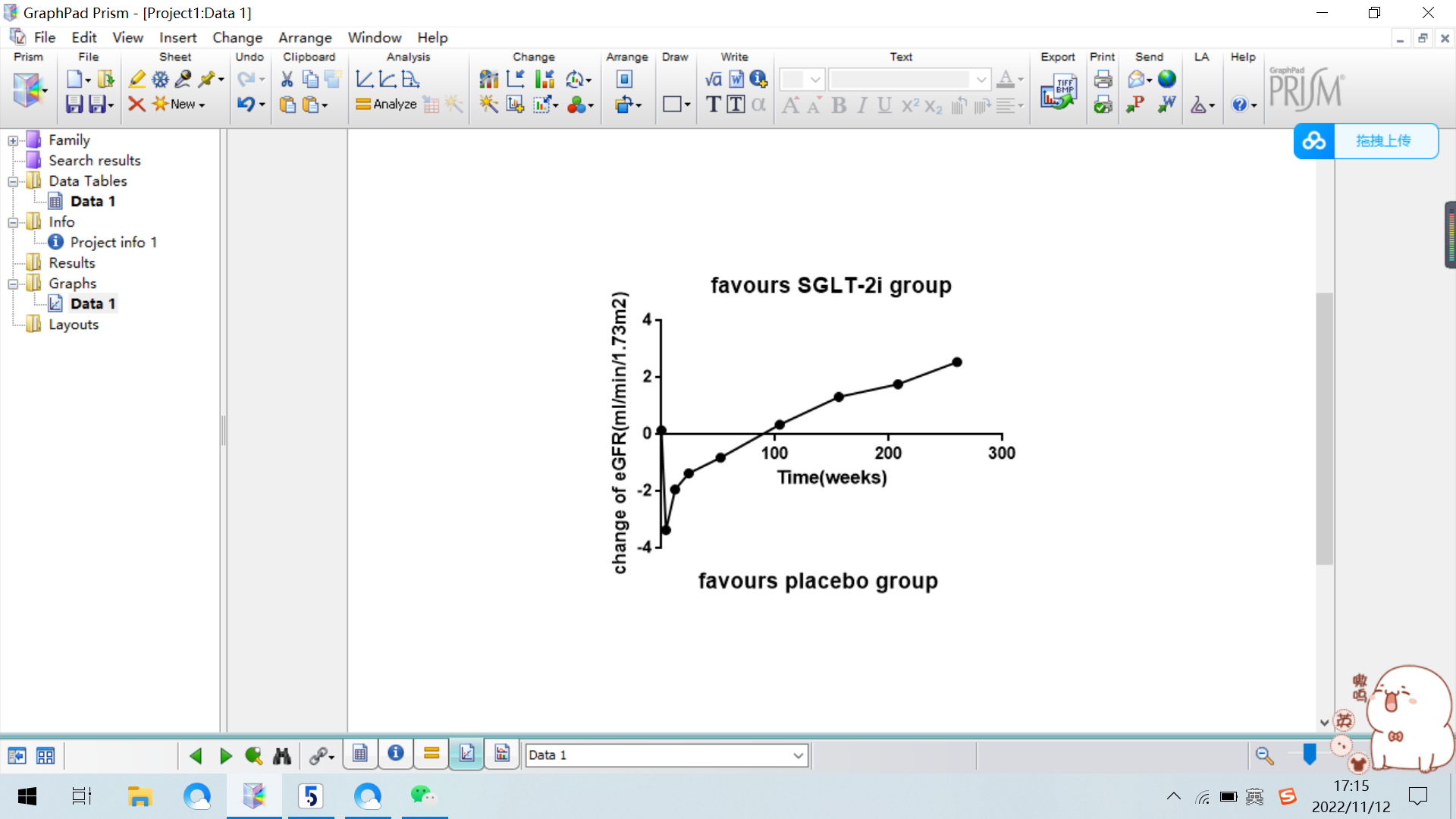


**Supplemental Fig 1.** The change of eGFR during the process of administration in the SGLT-2i group compared with the placebo group.


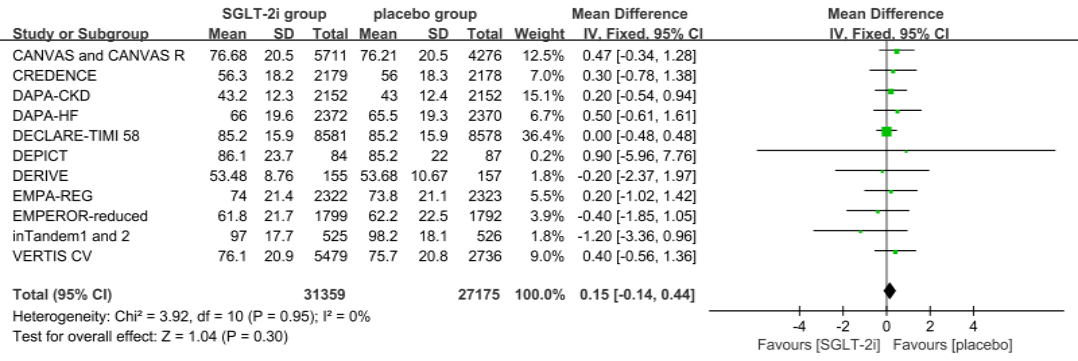


**Supplemental Fig 2.** The baseline eGFR of included 11 studies in the SGLT-2i group and the placebo group.


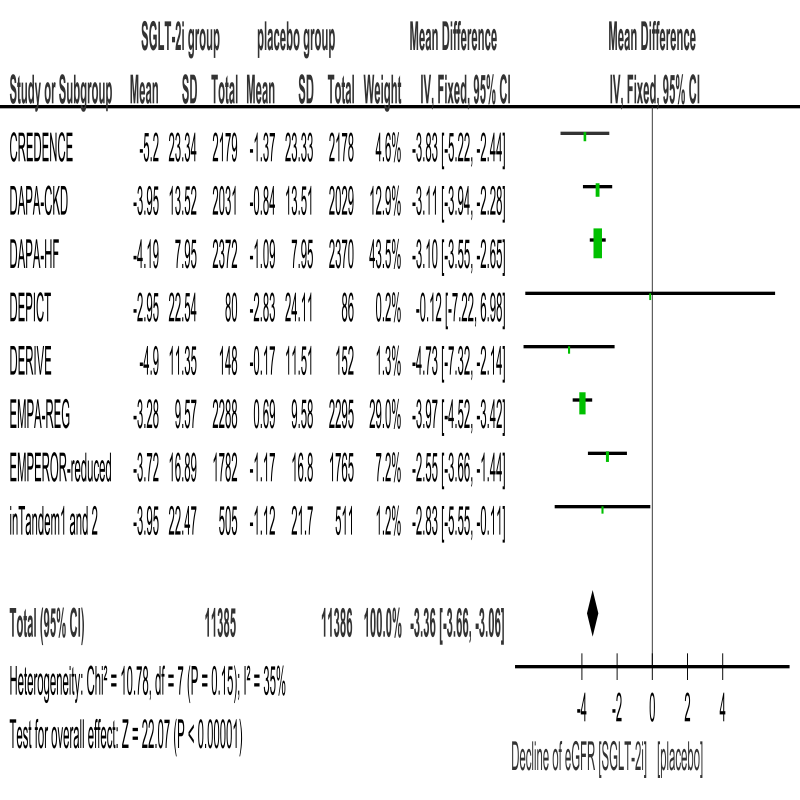


**Supplemental Fig 3.** The change of eGFR in the first 2-4 weeks after SGLT-2i used compared with placebo.


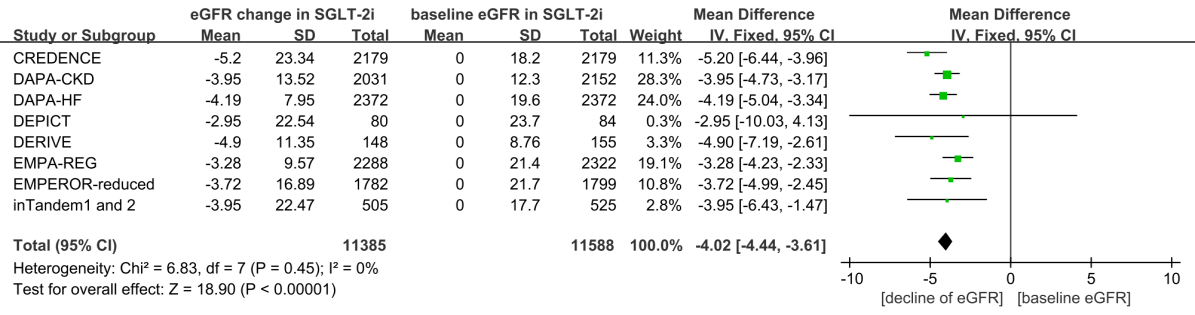


**Supplemental Fig 4.** The change of eGFR in the first 2-4 weeks compared with baseline eGFR in the SGLT-2i group.


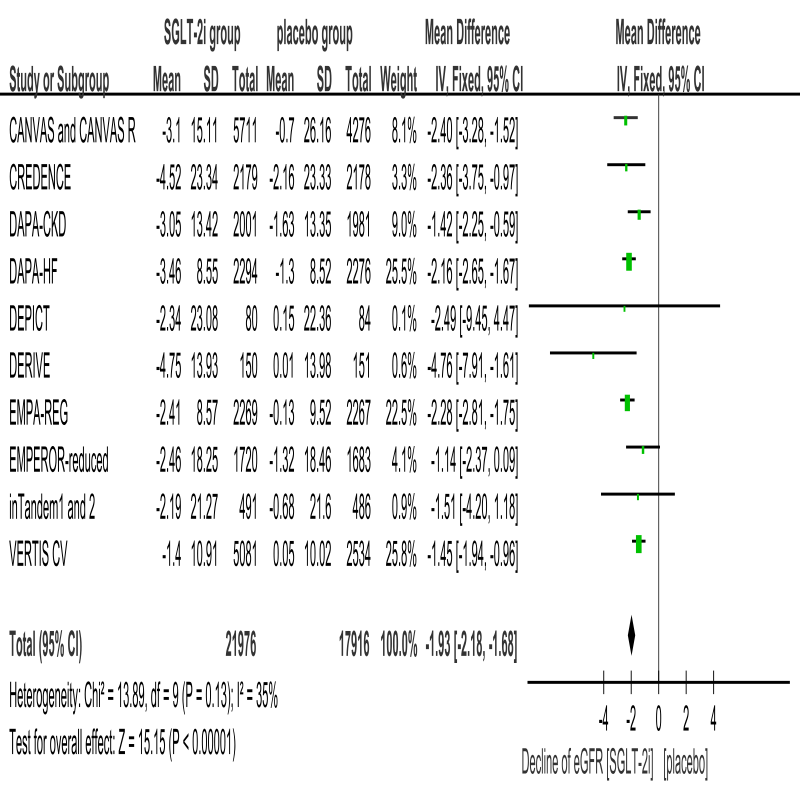


**Supplemental Fig 5.** The change of eGFR in the 12-18^th^ weeks after SGLT-2i used compared with placebo.


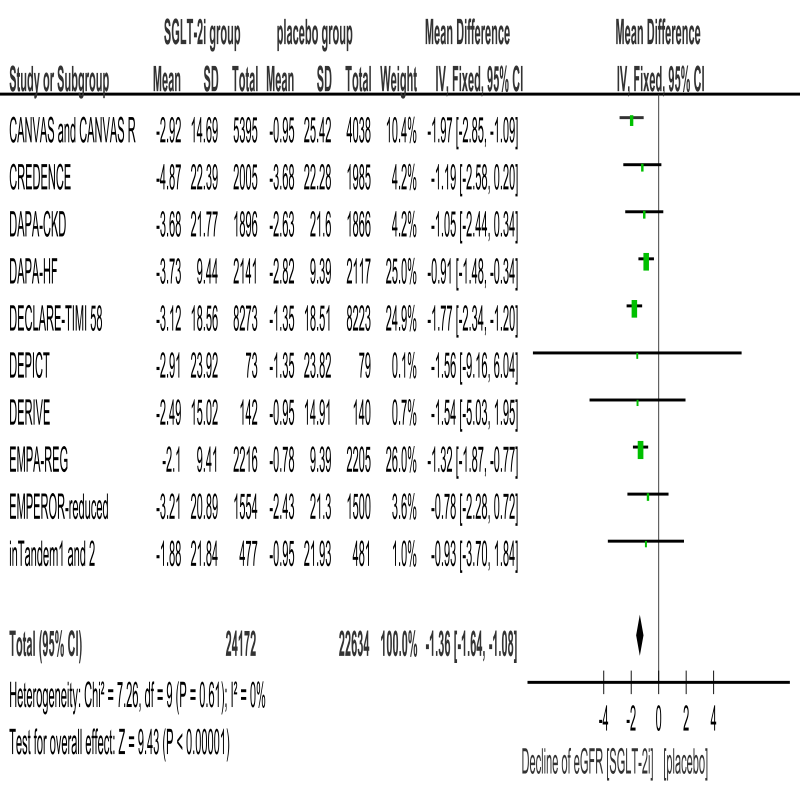


**Supplemental Fig 6.** The change of eGFR in the 24-32^th^ weeks after SGLT-2i used compared with placebo.


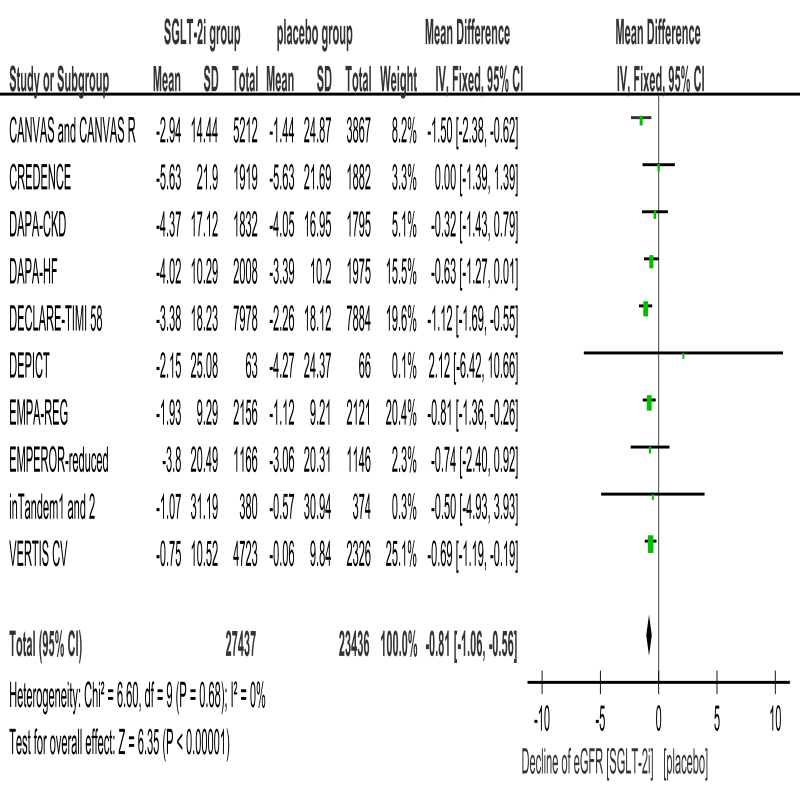


**Supplemental Fig 7.** The change of eGFR at the 52^th^ week (the first year) after SGLT-2i used compared with placebo.


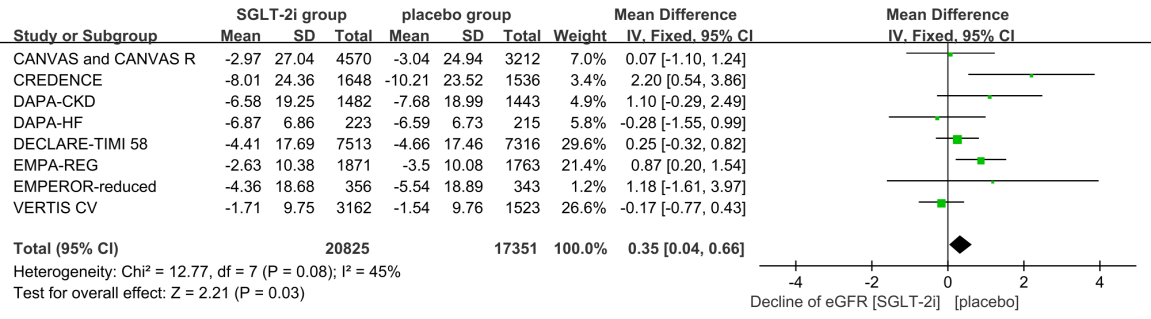


**Supplemental Fig 8.** The change of eGFR at the 104^th^ week (the second year) in the second year after SGLT-2i used compared with placebo.


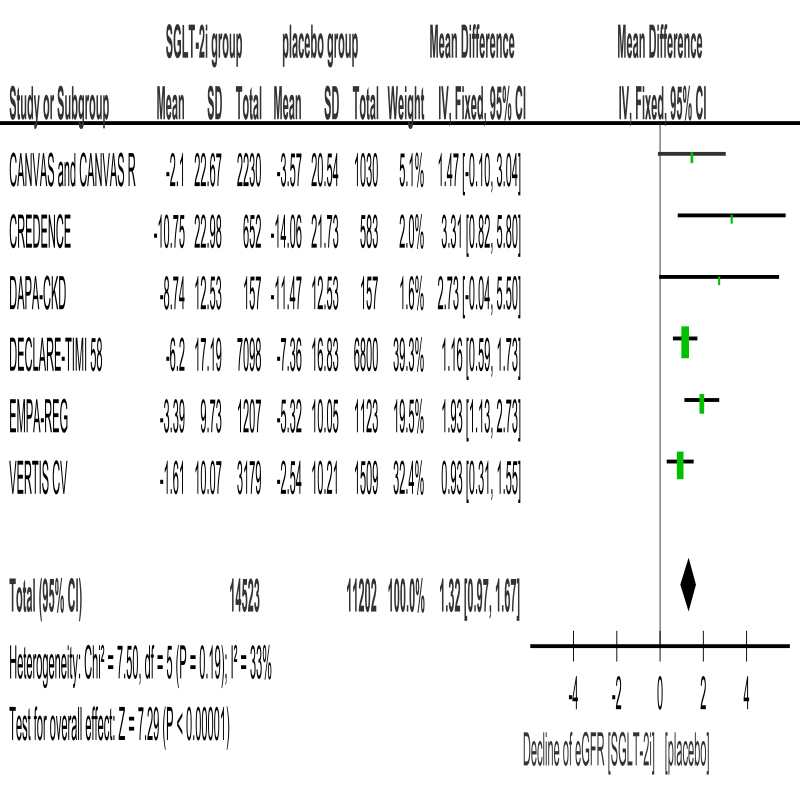


**Supplemental Fig 9.** The change of eGFR at the 156^th^ week (the third year) after SGLT-2i used compared with placebo.


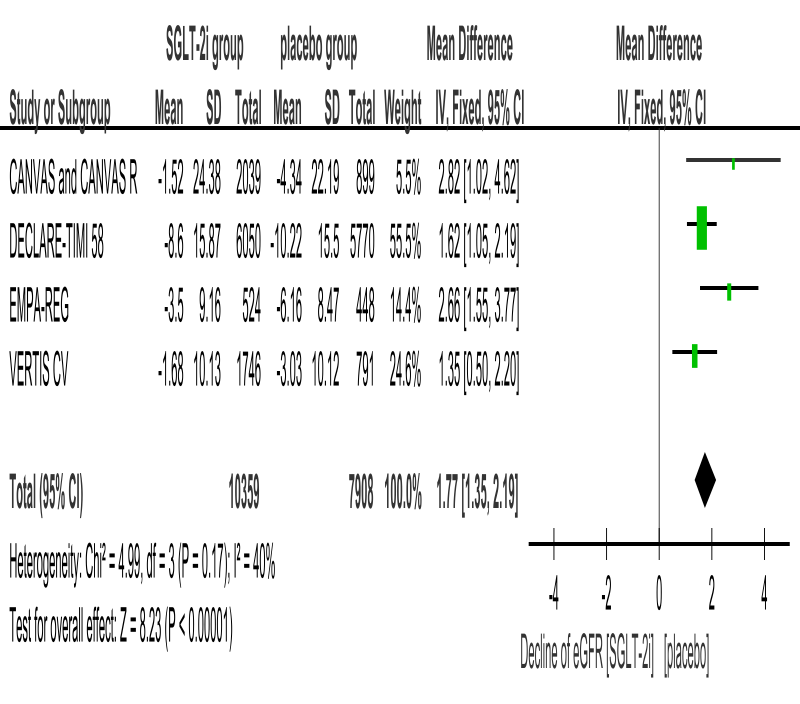


**Supplemental Fig 10.** The change of eGFR at the 208^th^ week (the fourth year) after SGLT-2i used compared with placebo.


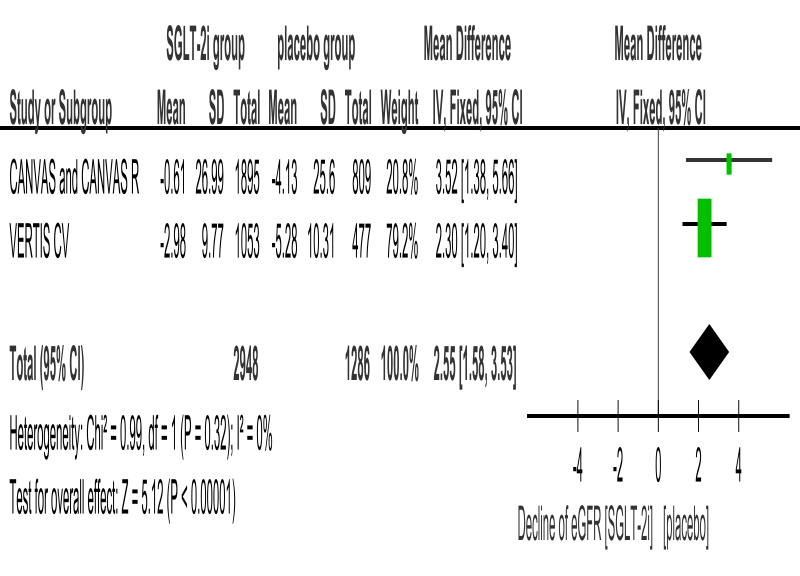


**Supplemental Fig 11.** The change of eGFR at the 260^th^ week (the fifth year) after SGLT-2i used compared with placebo.


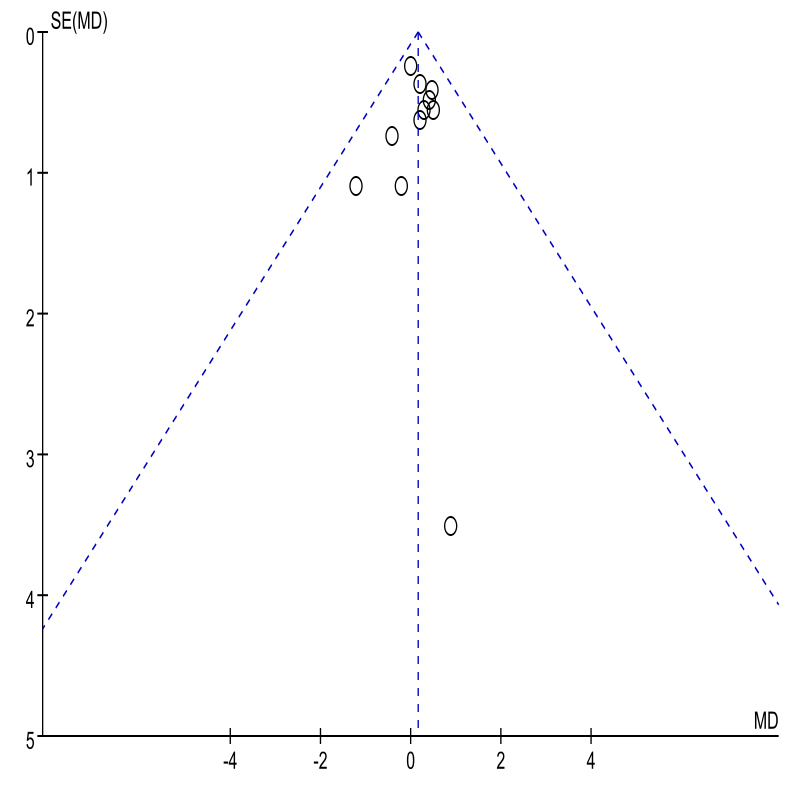


**Supplemental Fig 12.** The funnel plot of the included 11 studies.
